# Melatonin restores Muc2 depletion induced by *V. vulnificus* VvpM via melatonin receptor 2 coupling with Gαq

**DOI:** 10.1186/s12929-019-0606-x

**Published:** 2020-01-06

**Authors:** Young-Min Lee, Jong Pil Park, Young Hyun Jung, Hyun Jik Lee, Jun Sung Kim, Gee Euhn Choi, Ho Jae Han, Sei-Jung Lee

**Affiliations:** 1 Department of Pharmaceutical Engineering, Daegu Haany University, Hanuidae-ro 1, BioCenter, Suite 709, Gyeongsan-si, Gyeongsangbuk-do 38610 South Korea; 2 Department of Veterinary Physiology, College of Veterinary Medicine, Research Institute for Veterinary Science, and BK21 PLUS Program for Creative Veterinary Research Center, Seoul National University, Seoul, 08826 South Korea

**Keywords:** Melatonin, Melatonin receptor 2, Reactive oxygen species, Mucin, rVvpM, *V. vulnificus*

## Abstract

**Background:**

Melatonin (5-methoxy-N-acetyltryptamine), a hormone produced in the pineal gland, has a variety of biological functions as an antioxidant, but a functional role of melatonin in the regulation of intestinal mucin (Muc) production during bacterial infection has yet to be described in detail. In this study, we investigate the effects of melatonin during Muc2 repression elicited by the Gram-negative bacterium *V. vulnificus*.

**Methods:**

Mucus-secreting human HT29-MTX cells were used to study the functional role of melatonin during Muc2 depletion induced by the recombinant protein (r) VvpM produced by *V. vulnificus*. The regulatory effects of melatonin coupling with melatonin receptor 2 (MT_2_) on the production of reactive oxygen species (ROS), the activation of PKCδ and ERK, and the hypermethylation of the Muc2 promoter as induced by rVvpM were examined. Experimental mouse models of *V. vulnificus* infection were used to study the role of melatonin and how it neutralizes the bacterial toxin activity related to Muc2 repression.

**Results:**

Recombinant protein (r) VvpM significantly reduced the level of Muc2 in HT29-MTX cells. The repression of Muc2 induced by rVvpM was significantly restored upon a treatment with melatonin (1 μM), which had been inhibited by the knockdown of MT_2_ coupling with Gαq and the NADPH oxidase subunit p47 ^phox^. Melatonin inhibited the ROS-mediated phosphorylation of PKCδ and ERK responsible for region-specific hypermethylation in the *Muc2* promoter in rVvpM-treated HT29-MTX cells. In the mouse models of *V. vulnificus* infection, treatment with melatonin maintained the level of Muc2 expression in the intestine. In addition, the mutation of the *VvpM* gene from *V. vulnificus* exhibited an effect similar to that of melatonin.

**Conclusions:**

These results demonstrate that melatonin acting on MT_2_ inhibits the hypermethylation of the Muc2 promoter to restore the level of Muc2 production in intestinal epithelial cells infected with *V. vulnificus*.

## Background

The gastrointestinal mucus layer serves as the frontline defense barrier by promoting immune responses for the clearance of bacterial pathogens and by separating them from the intestinal epithelium [1]. Damage to the mucus layer facilitates intestinal epithelium-pathogen interactions and the invasion of pathogenic microorganisms [2]. The major component of this intestinal mucus layer is *O*-glycosylated mucin (Muc)2 in the human small intestine and colon. Muc2, produced by goblet cells, plays an important role as a physiological barrier via the formation of an enormous net-like mucus polymer barrier [2]. Indeed, *Muc2* deficiency in mice, which lack an inner mucus layer, causes the spontaneous development of inflammation, gross bleeding and increased paracellular permeability via unusual commensal bacteria colonization [3, 4]. Given that mucin plays a critical role in providing protection against the multiple inflammatory responses induced by invading pathogens and toxins, it is important to identify the factors that regulate *Muc2* gene expression, such as growth factors [5], transcription factors [6], and the methylation status [7].

*Vibrio (V.) vulnificus* is a rod-shaped anaerobic Gram-negative food pathogen that often causes acute inflammatory responses in the gut [8, 9]. Infection with *V. vulnificus* is cytotoxic to host cells, and its virulence is mediated by secreted cytotoxins and enzymes, such as VvhA, MARTX, VvpE, and VvpM [10–14]. A 55-kDa zinc-metalloprotease designated as *V. vulnificus* VvpM is considered to be major exoprotease that causes cytotoxic effects and an autophagic process affecting intestinal epithelial cells [13, 14]. We previously reported that VvpM induces the production of reactive oxygen species (ROS) and IL-1β coupled with necrotic macrophages via the transcriptional and epigenetic regulation of the inflammatory process [15]. However, the underlying cellular mechanisms of the VvpM involvement in the production of gastrointestinal mucin remain undescribed.

Melatonin (5-methoxy-N-acetyltryptamine) is a hormone produced in the pineal gland. It is readily available, produces few side effects, and is a relatively inexpensive substance. It is also a functional substance produced at various locations in body, including the skin, the lymphocytes and the gastrointestinal tract [16–18]. Melatonin mediates diverse effects through its cognate receptors, which include at least two members of the G protein-coupled receptor (GPCR) super-family, MT_1–2_, as well as MT_3_, while others may involve nuclear binding sites or may be receptor-independent [16]. It was previously shown that melatonin has a potential therapeutic effect on those with chronic obstructive pulmonary disease (COPD) by inhibiting mucin production [19]. In contrast, the underlying cellular mechanisms of melatonin that stimulate intestinal mucin production and the receptor specificity of intestinal epithelial cells involved in this process remain largely unknown. There are no previous reports related to the molecular mechanisms of the action of melatonin that which drives mucin production during a *V. vulnificus* infection.

In this study, therefore, we investigate the role of the melatonin signaling pathway in promoting gastrointestinal mucin production and evaluate its potential therapeutic effect against a *V. vulnificus* infection.

## Materials and methods

### Materials

Fetal bovine serum (FBS) was purchased from BioWhittaker (Walkersville, MO, USA). The following antibodies were purchased: PKCδ antibody (BD Biosciences, Franklin Lakes, NJ, USA); p-ERK, ERK, p-PKC, PKC and β-actin antibodies (Santa Cruz Biotechnology, Paso Robles, CA, USA); Muc2 antibody (Abcam, Cambridge, MA, USA); and horseradish peroxidase (HRP)-conjugated goat anti-rabbit and goat anti-mouse IgG antibodies (Jackson ImmunoResearch, West Grove, PA, USA). The 2′, 7′-dichlorofluorescein diacetate (CM-H_2_DCFDA) was obtained from Invitrogen (Carlsbad, CA, USA). Melatonin (Mel, 5-methoxy-N-acetyltryptamine) and N-acetyl-l-cysteine (NAC) were purchased from Sigma-Aldrich (St. Louis, MO, USA). All other reagents were of the highest purity commercially available and were used as received.

### Cells

Mucus-secreting human intestinal epithelial (HT29-MTX) and Caco-2 human intestinal epithelial cells were purchased from the American Type Culture Collection (ATCC, Manassas, VA, USA) and cultured at 37 °C in 5% CO_2_ in RPMI-1640 and DMEM containing 10% FBS and antibiotics (10 units/mL penicillin G and 10 μg/mL streptomycin). HT29-MTX cells have been used to study the adhesion and invasion of pathogens due to their physiologically relevant characteristics responsible for the formation of a mucus layer [20, 21]. Caco-2 cells were used as an alternative epithelial cell line to confirm the role of rVvpM in the Muc2 expression outcomes.

### Cell viability

A cell viability assay was conducted using the EZ-CYTOX cell viability kit (Dail-Lab Service, Seoul, Korea) according to the manufacturer’s instructions. Cells were cultured on 96-well culture plates. After incubation with rVvpM and melatonin, the EZ-CYTOX master mix was added to each well for 1 h. Cell viability was analyzed by measuring the absorbance at 450 nm.

### Bacterial strains, plasmids, and culture media

All *V. vulnificus* strains are isogenic and naturally resistant to polymyxin B (Table 1). Unless otherwise noted, *V. vulnificus* strains were grown in Luria Bertani (LB) media supplemented with 2.0% (wt/vol) NaCl (LBS) at 30 °C. All media components were purchased from Difco (Difco Laboratories Inc., Detroit, MI). *V. vulnificus* strains were grown to the mid-log phase (A_600_ = 0.500) corresponding to 2 × 10^8^ CFU/mL and were centrifuged at 6000×*g* for 5 min. The pellet was washed with phosphate-buffered saline (PBS) and adjusted to the desired colony-forming unit amount (CFU)/mL based on A_600_ as determined using a UV–VIS spectrophotometer (UV-1800, Shimadzu, Japan) to estimate the culture density.

**Table 1:** Plasmids and bacterial strains used in this study

Strain or plasmid	Relevant characteristics ^a^	Reference or source
Bacterial strains		
*V. vulnificus*		
M06–24/O	Clinical isolate; virulent; WT	Laboratory collection
ML05	MO6–24/O Δ*VvpM*; Km^r^; VvpM mut	Lee et al., 2013 [22]
*E. coli*		
DH5α	λ^−^ ϕ80d*lacZ*ΔM15 Δ (*lacZYA-argF*)*U169 recA1 endA1 hsdR17* (r_K_^−^ m_K_^−^) *supE44 thi-1 gyrA relA1*; plasmid replication	Laboratory collection
S17–1λ*pir*	λ-*pir* lysogen; *thi pro hsdR hsdM*^*+*^ *recA* RP4–2 Tc::Mu-Km::Tn7;Tp^r^ Sm^r^; host for π-requiring plasmids; conjugal donor	Simon et al., 1983 [23]
Plasmids		
pDM4	R6K γ *ori sacB*; suicide vector; *oriT* of RP4; Cm^r^	Milton et al., 1996 [24]
pRK415	IncP *ori*, broad-host-range vector; *oriT* of RP4; Tc^r^	Keen et al., 1988 [25]
pKK1535	pRK415 with *VvpM*; Tc^r^	This study

^a^ Km^r^, kanamycin resistant; Tp^r^, trimethoprim resistant; Sm^r^, streptomycin resistant; Cm^r^, chloramphenicol resistant; Tc^r^, tetracycline resistant

### Generation of the VvpM mutant and its complementation

Bacteria strains for the VvpM mutant and the complementation of the VvpM mutant were kindly provided by Prof. Sang Ho Choi (Seoul National University, Korea). Briefly, a deficient mutant in *VvpM* genes was generated by methods described previously [22, 26, 27]. For the generation of the VvpM mutant [24], *VvpM* was inactivated in vitro by the deletion of its open reading frame (ORF) using the PCR-mediated linker-scanning mutation method, as described in the literature [28]. Pairs of primers for VvpM-upF and -upR (for amplification of the 5′ amplicon) or VvpM-downF and -downR (for amplification of the 3′ amplicon) were designed and used (Additional file 1: Table S1). To complement the VvpM mutation process, ORF of *VvpM* was amplified from the genomic DNA of *V. vulnificus* MO6–24/O by PCR with the primer pair VvpM001F and VvpM001R (Additional file 1: Table S1) and then digested with BamHI and SacI. The amplified *VvpM* ORF was subcloned into the broad-host-range vector pRK415 [25] linearized with the same enzymes (Table 1) to result in pKK1535. The *E. coli* S17–1 *λpir, tra* strain [23] containing pKK1535 was used as a conjugal donor to the VvpM mutant. The plasmid pKK1535 was delivered into the VvpM mutant by conjugation, as described previously [28].

### Purification of the recombinant VvpM

The recombinant (r) VvpM protein [13, 14] was kindly provided by Prof. Kyu-Ho Lee (Sogang University, Korea). We assessed the rVvpM protein for LPS contamination using an endotoxin quantitation kit (Pierce® LAL Chromogenic Endotoxin Quantitation Kit, Thermo Fisher Scientific Inc. Waltham, MA, USA). The level of endotoxin in 100 pg/mL of rVvpM was less than 0.003 EU. Thus, we suggest that rVvpM is a purified recombinant protein containing a very low level of endotoxin and that it is suitable for our experiments in this study.

### Reverse transcription polymerase chain reaction (RT-PCR) and quantitative real-time PCR

Total RNA was extracted using the RNeasy Plus Mini Kit (Quiagen, Valencia, CA, USA). Reverse transcription (RT) was carried out with 3 μg of RNA using a Maxime RT premix kit (iNtRON Biotechnology, Sungnam, Korea). The cDNA (5 μl) for *MT*_*1*_, *MT*_*2*_, *Muc*2, and *β-actin* were amplified using the following primer pairs: *MT*_*1*_, 5′- TCCTGGTCATCCTGTCGGTGTATC-3′ and 5′-CTGCTGTACAGT TTGTCGTACTTG-3′; *MT*_*2*_, 5′-TCCTG GTGATCCTCTCCGTGCTCA-3′ and 5′-AGCCAGA TGAGGCAGATGTGCAGA-3′; *Muc*2, 5′-CAGCTCATCTCGTCCGTCTC-3′ and 5′-GCTGGCTGGTTTTCTCCTCT-3′; *β-actin*, 5′-AACCGCGAGAAGATGACC-3′ and 5′- AGCAG CCGTGGCCATCTC-3′. The real-time quantification of Muc2 was conducted using a Rotor-Gene 6000 real-time thermal cycling system (Corbett Research, New South Wales, Australia) with a QuantiMix SYBR kit (PhileKorea Technology, Daejeon, Korea) according to the manufacturer’s instructions. β-Actin was used as an endogenous control.

### Small interfering (si) RNA transfection

Cells were grown until 75% of the surface of the plate was covered and were then transfected for 36 h with ON-TARGETplus siRNAs mixed with six different siRNAs specific for *MT*_*2*_*, Gαq, Gαi, Gα12, NCF1, PKCδ,* and *ERK* (GE Dharmacon, Lafayette, CO, USA) or non-targeting (*nt*) siRNA as a negative control (GE Dharmacon, Lafayette, CO, USA) with the HiPerFect transfection reagent (QIAGEN, Valencia, CA, USA) according to the manufacturer’s instructions. The siRNA efficacy rates for *MT*_*2*_*, Gαq, Gαi, Gα12, NCF1, PKCδ,* and *ERK* as determined by Western blots were 68, 71, 70, 68, 66, 75, and 64%, respectively (data not shown).

### Intracellular reactive oxygen species (ROS) detection

2′,7′-dichlorofluorescein diacetate (CM-H_2_DCFDA) was used to detect the production of intracellular ROS. To quantify the intracellular ROS levels, cells treated with 10 mM DCF-DA were rinsed twice with ice-cold PBS and then scraped. A 100 μL cell suspension was loaded into a 96-well plate and examined using a luminometer (Victor3; Perkin-Elmer, MA, USA) and a fluorescent plate reader at excitation and emission wavelengths of 485 and 535 nm, respectively.

### Western blot analysis

Western blotting was performed as previously described with minor modifications [29]. Cells were harvested, washed twice with PBS, and lysed with a buffer (20 mM Tris [pH 7.5], 1 mM EDTA, 1 mM EGTA, 1% Triton X-100, 1 mg/mL aprotinin, and 1 mM phenylmethylsulfonyl fluoride [PMSF]) for 30 min on ice. The lysates were then cleared by centrifugation (22,250 x *g* at 4 °C for 30 min). Equal amounts of protein (20 μg) were resolved by 10 ~ 15% sodium dodecyl sulfate polyacrylamide gel electrophoresis (SDS-PAGE) and transferred to polyvinylidene fluoride (PVDF) membranes. The membranes were washed with TBST solution [10 mM Tris-HCl (pH 7.6), 150 mM NaCl, and 0.05% Tween-20], blocked with 5% skim milk for 1 h, and incubated with the appropriate primary antibody at 4 °C overnight. Each membrane was then washed and incubated with a horseradish peroxidase-conjugated secondary antibody for 2 h. The bands were visualized by enhanced chemiluminescence (Amersham Pharmacia Biotech Inc., Buckinghamshire, UK) and detected using a Bio-Rad ChemiDoc™ XRS+ system with images manipulated using Image Lab™ software #170–8265 (Bio-Rad, Hercules, CA, USA).

### Enzyme-linked immunosorbent assay (ELISA)

HT29-MTX cells plated on 60-mm culture dishes were grown in FBS-free media for 24 h and divided into groups according to the experimental protocol. In each case, the Muc2 concentration in the culture medium was quantified by an enzyme-linked immunosorbent assay (ELISA) (Elabscience Biotech Co., Ltd., Wuhan, Hubei, China) according to the manufacturer’s instructions.

### Immunofluorescence and immunohistochemical analysis

Either HT29-MTX cells or ileum frozen sections were fixed in 4% paraformaldehyde in PBS for 10 min at room temperature, permeabilized in 0.1% Triton X-100 in PBS for 5 min, and blocked in PBS containing 5% (v/v) normal goat serum (NGS) for 30 min at room temperature. Samples were then stained with primary antibody overnight at 4 °C. Following three washes with PBS, the samples were incubated with Alexa 488-conjugated goat anti-rabbit/mouse IgM (Invitrogen Co., Carlsbad, CA, USA) and counterstained with PI in PBS containing 5% (v/v) NGS for 2 h. After washing with PBS, the samples were mounted on slides and visualized with an Olympus FluoView™ 300 confocal microscope with a 400x objective lens. For an immunohistochemical analysis, ileum frozen tissues incubated with primary antibody overnight at 4 °C were treated with a biotinylated secondary antibody solution (Vectastain Elite ABC kit, Vector Laboratories, CA, USA) for 1 h at room temperature. Sections were washed with PBS, incubated in the ABC reagent for 1 h at room temperature, washed again, and incubated in a peroxidase solution. The sections were then counterstained with hematoxylin, dehydrated, and coverslipped. Images were acquired using an Axioskop 2 plus microscope equipped with an AxioCam MRc5 CCD camera (Zeiss). Other samples were subjected to hematoxylin and eosin (H&E) staining for histological examinations.

### Methylation analysis

Genomic DNA from the HT29-MTX cells was prepared with the QIAamp DNA Mini Kit (Qiagen, Valencia, CA, USA). The extracted DNA was treated with sodium bisulfite using an EzWay™ DNA methylation detection kit according to the manufacturer’s instructions (KOMA BIOTECH, Seoul, Korea). The methylation status of the *Muc2* gene in the HT29-MTX cells was determined by a methyl-specific PCR (MSP) analysis. We conducted MSP for the *Muc2* gene promoter at two CpG sites (− 274 and − 193) which play functional roles in the regulation of *Muc2* expression, as described previously [30]. The primer sequences for each CpG site of the *Muc2* promoter and the size of products are summarized in Additional file 1: Table S2.

### Mouse model and lethality rate

All animal procedures were performed following the National Institutes of Health Guidelines for the Humane Treatment of Animals, with approval from the Institutional Animal Care and Use Committee of Seoul National University (SNU-140108-4). Seven-week-old male ICR mice (*n* = 8) received an i.g. inoculation of boiled *V. vulnificus* (Cont), *V. vulnificus* (WT), WT, VvpM mutant, or VvpM complement at 1.3 × 10^9^ CFU/mL and after 16 h were sacrificed. To evaluate the functional role of melatonin, the mice had been given an oral administration of melatonin (10 mg/kg) once a day for 7 days prior to their infection of WT for 16 h, which is the minimum duration for intestinal colonization by WT [31]. The ileum tissue was embedded in O.C.T. compound and stored at − 70 °C. Samples were then cut into 6-μm-thick frozen sections. To determine the mouse lethality rate, seven-week-old mice (*n* = 10) given 250 mg/kg of iron dextran received an oral administration of melatonin (10 mg/kg) daily for 7 days prior to i.p. inoculation with boiled *V. vulnificus* (Cont) and *V. vulnificus* (WT) at 1.2 × 10^2^ CFU/ml, with the survival rate of the mice then recorded for 24 h.

### Histologic damage score

Histological parameters were determined in a blinded fashion by two experienced gastrointestinal pathologists, as previously described [32], with some modification. Briefly, scores were assigned as follows: 0 = no damage (normal); 1 = slight submucosal and/or lamina propria separation (mild); 2 = moderate separation of the submucosa and/or lamina propria and/or edema in the submucosa and muscular layers (moderate); 3 = severe separation of the submucosa and/or lamina propria and/or severe edema in the submucosa and muscular layers with regional villous sloughing (severe); or 4 = loss of villi and necrosis (necrosis). Intermediate scores of × 0.0 or × 0.5 were also used to assign the degree of intestinal damage more precisely.

### Statistical analysis

Results are expressed as means ± standard errors (S.E.). All experiments were analyzed by ANOVA, followed in some cases by a comparison of treatment means with a control using the Bonferroni-Dunn test. Differences were considered statistically significant at *P* < 0.05.

## Results

### Melatonin regulates the level of Muc2 in intestinal epithelial cells treated with rVvpM

**Fig. 1:**
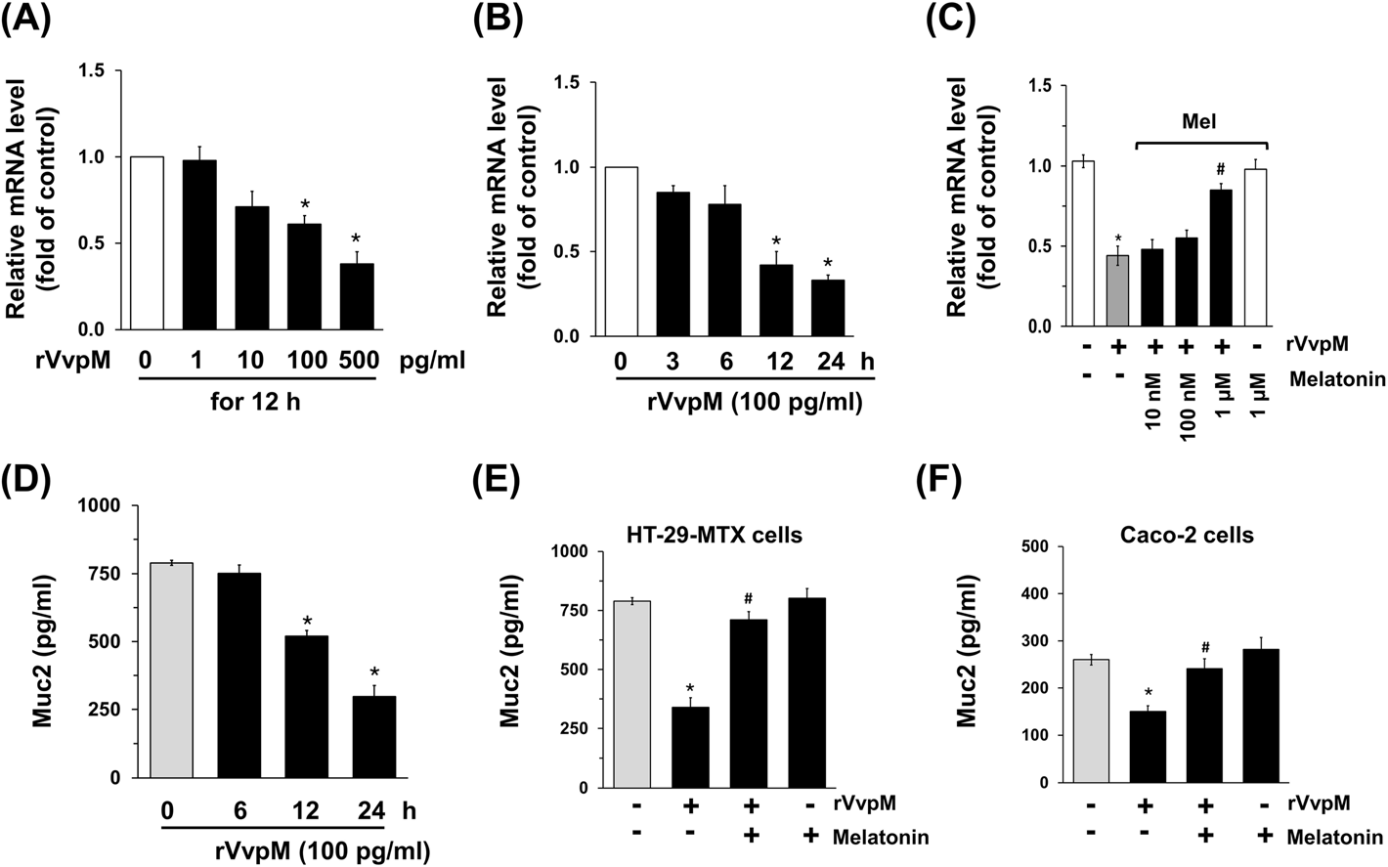
Melatonin regulates the level of Muc2 in intestinal epithelial cells treated with rVvpM. Dose (**a**) and time (**b**) responses of rVvpM in *Mucin (Muc)*2 mRNAs of HT29-MTX cells are shown. Data represent means ± S.E. *n* = 5. *, *p* < 0.05 vs. 0 pg/mL. **c** Cells were treated with melatonin at various concentrations (10 nM ~ 1 μM) for 30 min prior to rVvpM (100 pg/mL) exposure for 12 h. Expression of *Muc2* is shown. Data represent means ± S.E. *n* = 4. *, *p* < 0.05 vs. vehicle. ^*#*^, *p* < 0.01 vs. rVvpM alone. **d** Time responses of 100 pg/mL of rVvpM in Muc2 ELISA assay are shown. Error bars represent the means ± S.E. *n* = 4. *, *p* < 0.01 vs. 0 h. HT29-MTX (**e**) and Caco-2 human intestinal epithelial cells (**f**) were incubated with melatonin (1 μM) for 30 min prior to rVvpM exposure for 24 h. The level of Muc2 production was quantified by ELISA. Data represent means ± S.E. *n* = 4. *, *p* < 0.01 vs. vehicle. ^*#*^, *p* < 0.05 vs. rVvpM alone

To find the functional role of melatonin in mucin production, we used mucus-secreting human HT29-MTX cells, which form a homogeneous population of polarized goblet cells. These types of cells have been widely used in the field of mucin-related research [21]. Previous work has shown that *Muc2* mRNA is most abundant in HT29-MTX cells, followed by *Muc6*, whereas the expression levels of *Muc1*, *Muc3*, *Muc4*, and *Muc5* are relatively low [33]. To evaluate the role of *Vvp*M in the regulation of *Muc2* expression levels, HT29-MTX cells were exposed to various concentrations (0~500 pg/mL) of the recombinant protein (r) VvpM for 12 h. Compared to the cells with no treatment, rVvpM treatments from 100 to 500 pg/mL significantly inhibited the *Muc2* expression levels of HT29-MTX cells (Fig. 1a). A decrease in *Muc2* expression was observed after 12 h of incubation with 100 pg/mL of rVvpM (Fig. 1b). In addition, we found that 100 pg/mL of rVvpM did not have cytotoxic effects on HT-29 MTX cells for 24 h (Additional file 1: Figure S1A), suggesting that *Muc2* repression induced by rVvpM is not affected by a decreased number of viable cells. To determine the molecular mechanisms by which melatonin regulates intestinal mucin production, cells were treated with melatonin at various concentrations (10 nM–1 μM) 30 min prior to rVvpM (100 pg/mL) for 12 h. Interestingly, the inhibitory effect of rVvpM on *Muc*2 expression was silenced by a pretreatment with melatonin at a concentration of 1 μM (Fig. 1c). We also explored the ability of rVvpM to regulate Muc2 production in an ELISA assay. In contrast to the control, 100 pg/mL of rVvpM evoked a substantial reduction of Muc2 secretion for 24 h (Fig. 1d), with this decrease significantly blocked by a pretreatment with 1 μM of melatonin (Fig. 1e). Melatonin itself did not affect Muc2 production. These results suggest that rVvpM regulates the expression and secretion of Muc2 and that the protective effect of melatonin on *V. vulnificus* infection is related to the blockage of mucin repression caused by rVvpM. Similar effects were seen in Caco-2 cells (Fig. 1f), suggesting that the functional roles of rVvpM and melatonin are reproducible in other types of human epithelial cells. In addition, a decrease in Muc2 protein levels was observed after 12 h of incubation with 100 pg/ml of rVvpM. This result indicates that Muc2 repression induced by rVvpM in a culture supernatant is significantly influenced by the levels of Muc2 mRNA and protein (Additional file 1: Figure S1B). We explored the ability of melatonin to regulate Muc2 repression as induced by rVvpE, which is known to ameliorate Muc2 production as another toxin of *V. vulnificus*. In contrast to the control, 50 pg/ml of rVvpE evoked a substantial reduction of Muc2 secretion, whereas the repression activity of rVvpE on Muc2 was blocked by the pretreatment with melatonin (Additional file 1: Figure S2). This result indicates that the functional role of melatonin is reproducible in other types of toxins produced by *V. vulnificus*.

### Melatonin triggers the MT_2_ receptor-mediated signaling pathway to regulate ROS production induced by rVvpM

**Fig. 2:**
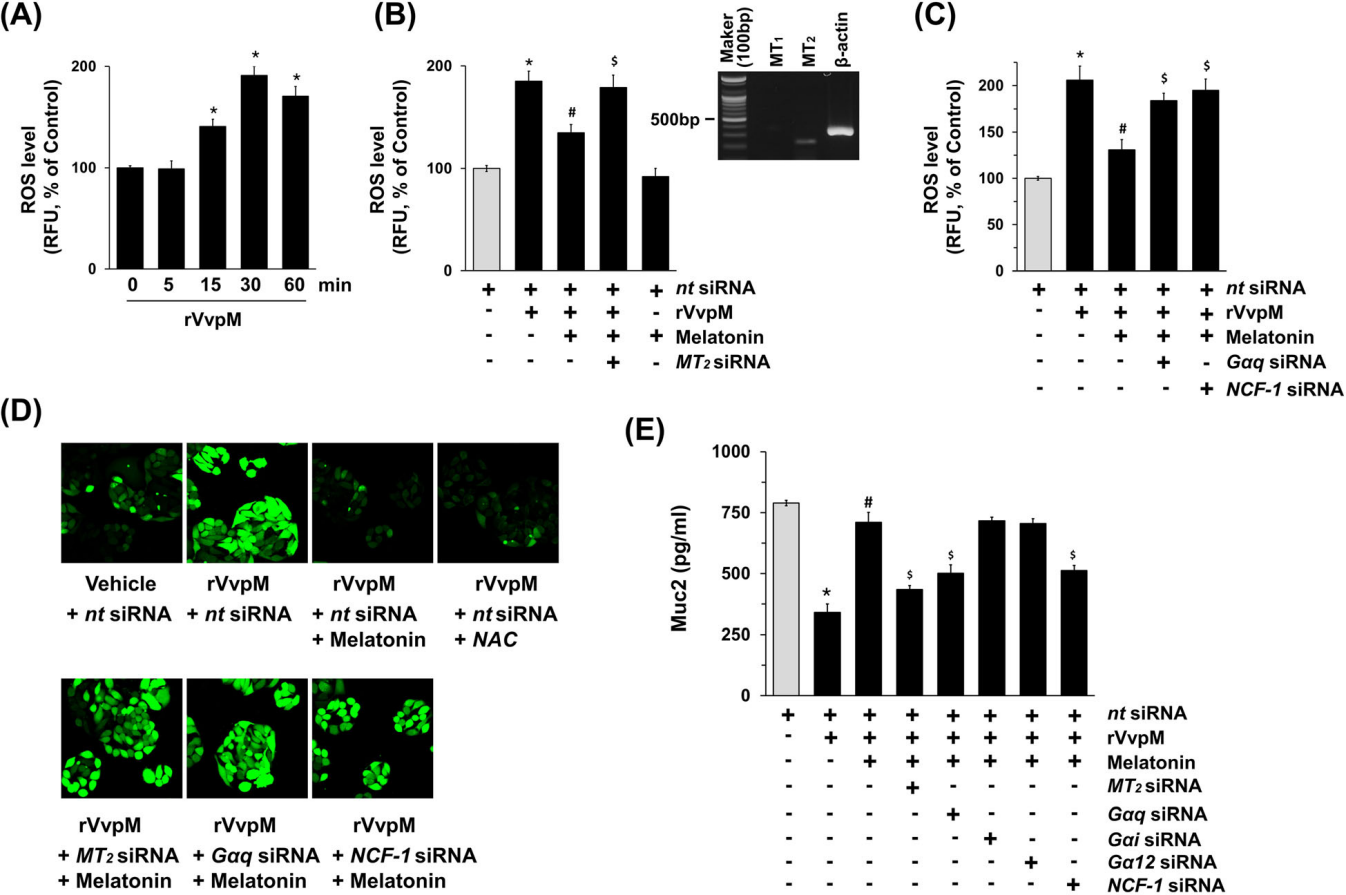
Melatonin triggers the MT_2_ receptor-mediated signaling pathway to regulate ROS production induced by rVvpM. **a** Time responses of rVvpM in ROS production are shown. Data represent means ± S.E. *n* = 4. *, *p* < 0.01 vs. 0 min. RFU, relative fluorescence units. **b** Cells transfected with siRNA for non-targeting (*nt*) control or *MT*_*2*_ for 24 h were incubated with melatonin (1 μM) for 30 min prior to rVvpM exposure for 30 min. The level of ROS production is shown. Inset shows the expression of MT_1_ and MT_2_ on total RNA of HT29-MTX cells. Data represent means ± S.E. *n* = 4. *, *p* < 0.001 vs. *nt* siRNA. ^*#*^, *p* < 0.01 vs. rVvpM + *nt* siRNA. ^$^, *p* < 0.01 vs. rVvpM + melatonin+ *nt* siRNA. **c** Cells transfected with siRNA for *Gαq* or *NCF-1* for 24 h were incubated with melatonin (1 μM) for 30 min prior to rVvpM exposure for 30 min. The level of ROS production is shown. Data represent means ± S.E. *n* = 4. *, *p* < 0.01 vs. *nt* siRNA. ^*#*^, *p* < 0.01 vs. rVvpM + *nt* siRNA. ^$^, *p* < 0.05 vs. rVvpM + melatonin+ *nt* siRNA. **d** Cells transfected with siRNA for *MT*_*2*_, *Gαq,* or *NCF-1* for 24 h were incubated with melatonin (1 μM) or NAC (10 μM) for 30 min prior to rVvpM exposure for 30 min. ROS production (green) was visualized by confocal microscopy. Scale bars, 100 μm. *n* = 3. **e** Cells transfected with siRNA for *MT*_*2*_, *Gαq, Gαi, Gα12* or *NCF-1* for 24 h were incubated with melatonin (1 μM) for 30 min prior to rVvpM exposure for 24 h. The level of Muc2 production was quantified by ELISA. Data represent means ± S.E. *n* = 4. *, *p* < 0.01 vs. *nt* siRNA. ^*#*^, *p* < 0.05 vs. rVvpM + *nt* siRNA. ^$^, *p* < 0.05 vs. rVvpM + melatonin+ *nt* siRNA

To determine the molecular mechanisms related to mucin regulation, we conducted a closer examination of the roles of rVvpM and melatonin in the production of reactive oxygen species (ROS), which are crucial for controlling both microbial infections and melatonin functions. A significant increase in the ROS level was observed, at 15 and 60 min after incubation with 100 pg/mL of rVvpM (Fig. 2a), though the increase at 30 min could be blocked by 1 μM of melatonin (Fig. 2b). In addition, we determined the levels of production of ROS upon long-term exposure to rVvpM. We found that rVvpM significantly stimulates the production of ROS for 12 h compared to cells with no treatment (Additional file 1: Figure S3A). In order to examine the mechanism causing these effects by melatonin, we determined the expression levels of melatonin receptors in HT29-MTX cells. As shown in the inset in Fig. 2b, we found that HT29-MTX cells harbor MT_2_, but not MT_1_. Moreover, the inhibitory effect of melatonin on ROS production was attenuated by the knockdown of *MT*_*2*_ expression with *MT*_*2*_ siRNA in HT29-MTX cells treated with rVvpM (Fig. 2b), suggesting that melatonin acting on MT_2_ is crucial for controlling *V. vulnificus* infections. In addition, the knockdown of genes for the heterotrimeric Gαq protein and the NADPH oxidase including p47 ^phox^ (NCF-1) significantly abrogated an anti-oxidative effect of melatonin (Fig. 2c). These results indicate that melatonin inhibits ROS production via MT_2_ coupling with Gαq and NCF-1. The anti-oxidative effect of melatonin via MT_2_ coupling with Gαq and NCF-1 was further visualized by staining the HT29-MTX cells with a fluorescent dye, CM-H_2_DCFDA (Fig. 2d). The production of ROS by rVvpM was significantly blocked by the treatment with melatonin as well as an antioxidant, N-acetylcysteine (NAC) (Fig. 2d, top panel). In addition, a pre-treatment with siRNAs for *MT*_*2*_, *Gαq* and *NCF-1* significantly blocked the anti-oxidative effect of melatonin in rVvpM-treated HT-29-MTX cells (Figs. 2d, bottom panel). Consistent with this, the protective effect of melatonin during Muc2 repression elicited by rVvpM was significantly silenced by the knockdown of *MT*_*2*_, *Gαq* and *NCF-1* (Fig. 2e). The silencing of *Gαi* and *Gα12* with siRNAs did not affect Muc2 recovery as mediated by Mel, ruling out the roles of Gαi and Gα12. These results indicate that the protective effect of melatonin on ROS production is related to the blockage of mucin repression caused by rVvpM and that melatonin triggers the MT_2_ receptor-mediated signaling pathway.

### Melatonin inhibits the PKCδ/ERK pathway activated by rVvpM

**Fig. 3:**
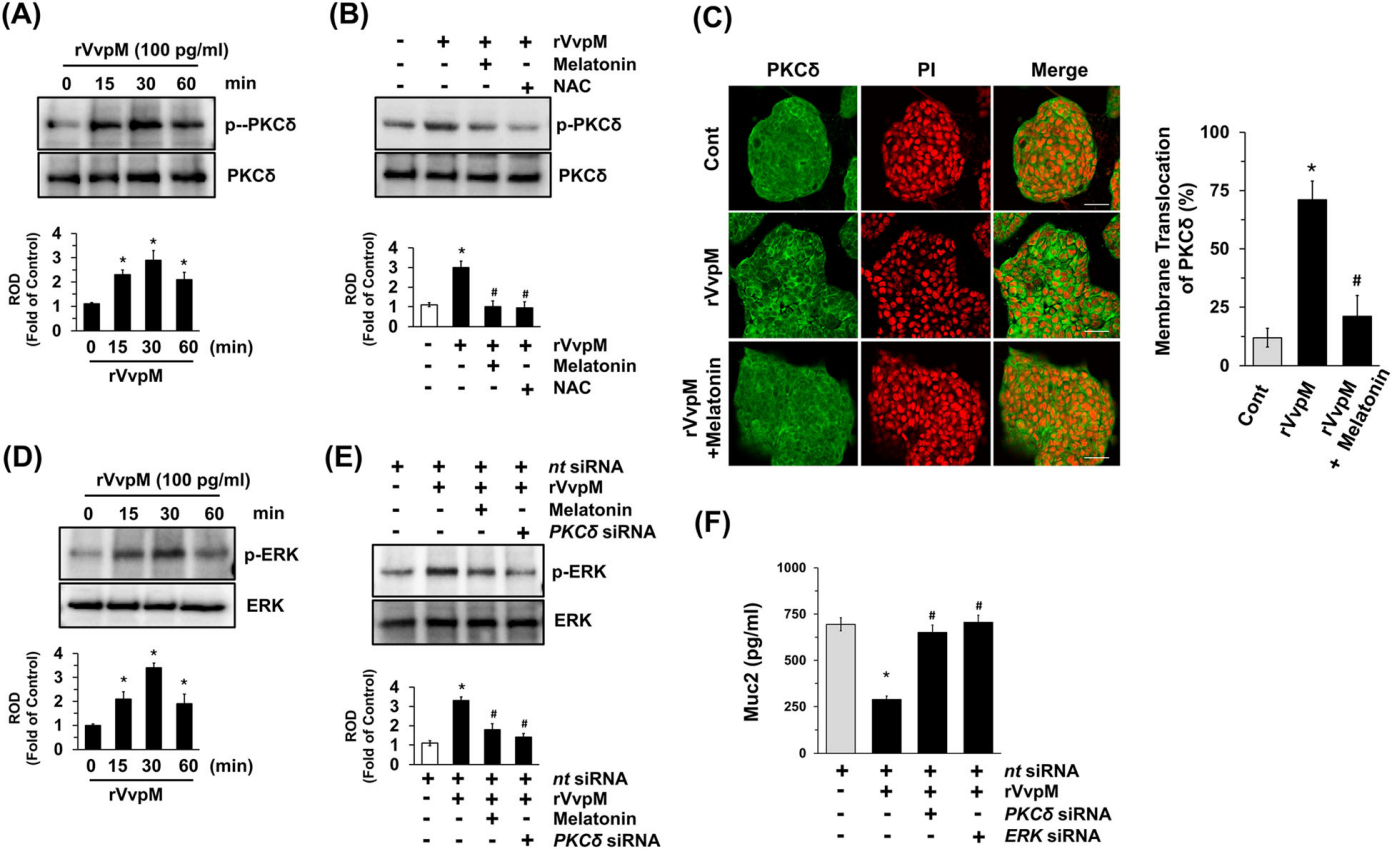
Melatonin inhibits PKCδ/ERK pathway activated by rVvpM. **a** Time responses of rVvpM in phosphorylation of PKCδ are shown. Data represent means ± S.E. *n* = 4. *, *p* < 0.001 vs. 0 min. ROD, relative optical density. **b** Phosphorylation of PKCδ in cells treated with melatonin (1 μM) or with NAC (10 μM) for 30 min prior to rVvpM exposure for 30 min is shown. Data represent means ± S.E. *n* = 4. *, *p* < 0.01 vs. vehicle. ^*#*^, *p* < 0.01 vs. rVvpM alone. **c** Membrane translocation of PKCδ (green) was determined by confocal microscopy. Propidium iodide (PI) was used for nuclear counterstaining (red) (right panel). The cell numbers showing membrane translocalization of PKCα per microscopic filed were directly counted and converted to a percentage by multiplying by 100. Ten random fields per coverslip were counted (left panel). Data represent the mean ± S.E. Scale bars, 100 μm. *n* = 3. **d** Time responses of rVvpM in phosphorylation of ERK are shown. Data represent means ± S.E. *n* = 4. *, *p* < 0.05 vs. 0 min. **e** Phosphorylation of ERK in cells treated with melatonin (1 μM) for 30 min prior to rVvpM exposure for 30 min is shown. Cells transfected with *PKCδ* siRNA for 24 h were also incubated with rVvpM for 30 min. Data represent means ± S.E. *n* = 4. *, *p* < 0.05 vs. *nt* siRNA. ^*#*^, *p* < 0.01 vs. rVvpM + *nt* siRNA. **f** Cells transfected with siRNA for *PKCδ* or *ERK1/2* for 24 h were incubated with rVvpM for 24 h. The level of Muc2 production was quantified by ELISA. Data represent means ± S.E. *n* = 4. *, *p* < 0.01 vs. *nt* siRNA. ^*#*^, *p* < 0.01 vs. rVvpM + *nt* siRNA

Many bacterial stimuli regulate the PKC and MAPK pathways, which are important candidates as downstream mediators of ROS. It was previously reported that PKCδ is one of PKC isotypes responsible for Muc2 repression induced by *V. vulnificus* [33]. As expected, treatment with rVvpM significantly induced the phosphorylation of PKCδ between 15 and 60 min (Fig. 3a), with the increase at 30 min blocked by a treatment with melatonin as well as an antioxidant, NAC (Fig. 3b). The membrane translocation of PKCδ was further confirmed by immunofluorescence staining in rVvpM-treated HT29-MTX cells (Fig. 3c). However, melatonin significantly blocked PKCδ activation induced by rVvpM. We then determined how rVvpM is linked to the activation of MAPKs, which are interesting candidates as downstream mediators of PKC in the regulation of mucin production [33]. rVvpM increased the phosphorylation of ERK between 15 and 30 min (Fig. 3d). In addition, the phosphorylation of ERK evoked by a treatment with rVvpM was markedly inhibited by a treatment with melatonin and the knockdown of *PKC*δ by siRNA (Fig. 3e). We also determined the levels of the phosphorylation of PKCδ and ERK during long-term exposure to rVvpM. Significant increases in the phosphorylation of PKCδ and ERK appeared after incubation with 100 pg/ml for 6 h compared to a control (Additional file 1: Figure S3B-C). These results provide strong evidence of the time differential between the activation of the signal cascade that leads to Muc2 repression. To provide more evidence of the involvement of PKCδ and ERK, we studied whether the knockdown of *PKC*δ and *ERK* regulates mucin repression induced by rVvpM (Fig. 3f). The silencing of *PKC*δ and *ERK* by siRNAs had a significant inhibitory effect on mucin repression in rVvpM-treated HT29-MTX cells. These results indicate that the inhibitory effect of melatonin on the phosphorylation of both PKCδ and ERK is related to the blockage of mucin repression caused by rVvpM.

### Regulatory effect of melatonin on the hypermethylation of the *Muc2* promoter induced by rVvpM

**Fig. 4:**
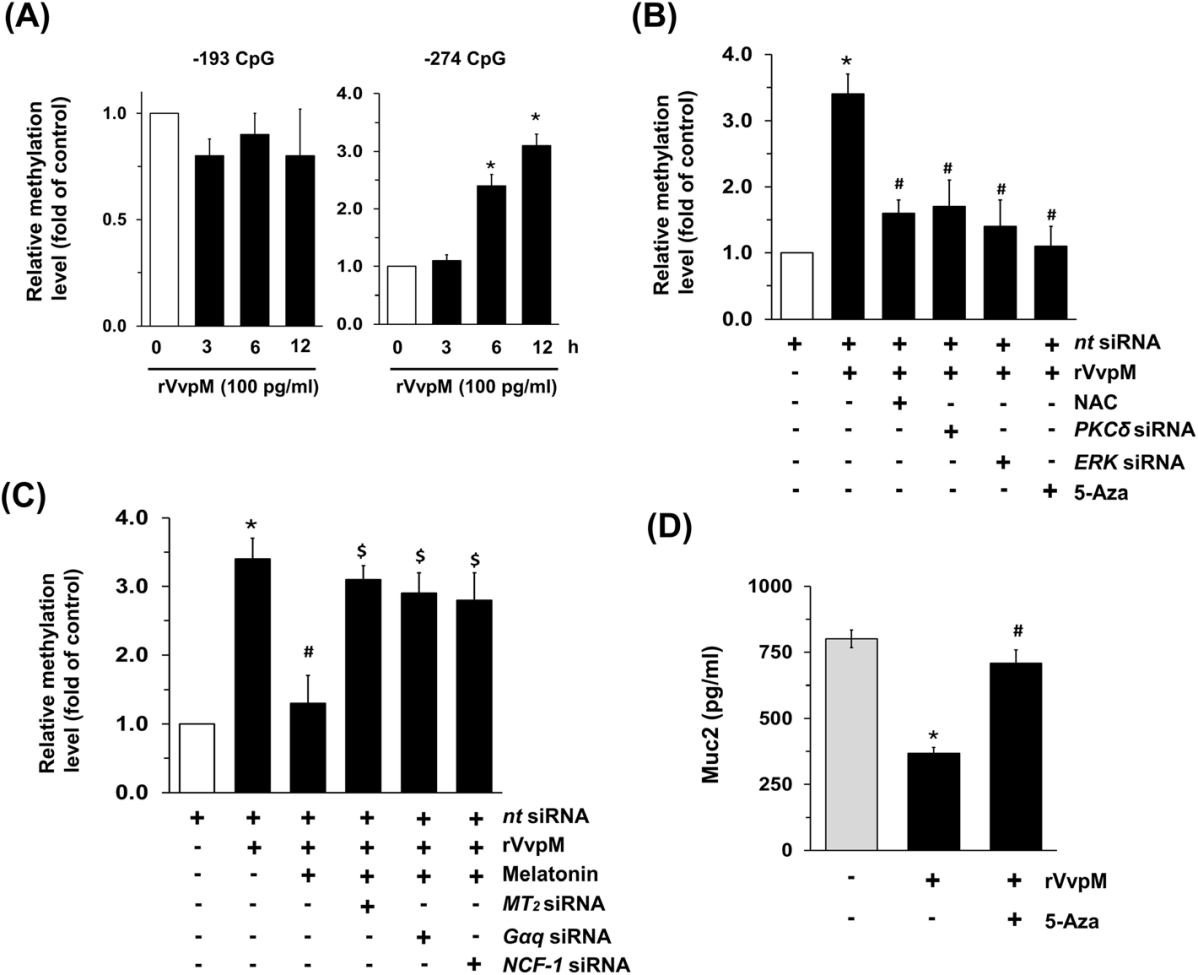
Regulatory effect of melatonin on hypermethylation of *Muc2* promoter induced by rVvpM. **a** Genomic DNA treated with rVvpM for 12 h was prepared. Time responses of rVvpM in methylation status of *Muc2* gene at − 274 and − 193 CpG sites were determined by methyl-specific PCR (MSP) analysis. The relative level of *Muc2* methylation is shown, compared to the unmethylated form. Data represent means ± S.E. *n* = 4. *, *p* < 0.05 vs. 0 h. **b** Cells were transfected with siRNA for *PKCδ* or *ERK1/2* for 24 h, or pre-treated with NAC (10 μM) or 5-aza (1 μM) for 30 min prior to rVvpM exposure for 12 h. The relative level of *Muc2* methylation is shown, compared to the unmethylated form. Data represent means ± S.E. *n* = 4. *, *p* < 0.05 vs. *nt* siRNA. ^*#*^, *p* < 0.01 vs. rVvpM + *nt* siRNA. **c** Cells transfected with siRNA for *MT*_*2*_, *Gαq,* or *NCF-1* for 24 h were incubated with melatonin (1 μM) for 30 min prior to rVvpM exposure for 12 h. The relative level of *Muc2* methylation is shown, compared to the unmethylated form. Data represent means ± S.E. *n* = 4. *, *p* < 0.05 vs. *nt* siRNA. ^*#*^, *p* < 0.01 vs. rVvpM + *nt* siRNA. ^$^, *p* < 0.05 vs. rVvpM + melatonin+ *nt* siRNA. **d** Cells were pre-treated with 5-aza (1 μM) for 30 min prior to rVvpM exposure for 24 h. The level of Muc2 production was quantified by ELISA. Data represent means ± S.E. *n* = 4. *, *p* < 0.01 vs. vehicle. ^*#*^, *p* < 0.05 vs. rVvpM alone

Because ERK is known to affect gene methylation directly for epigenetic alteration [34], the methylation status following a treatment with rVvpM was determined by means of methyl-specific PCR (MSP), after which the relative value of CpG methylation compared to an unmethylated form in the *Muc2* promoter was quantified via a real-time PCR analysis. We attempted to analyze the methylation status of the *Muc2* gene promoter containing two CpG sites, − 193 and − 274, which are critical CpG sites responsible for the repression of *Muc2* expression [30]. As shown in Fig. 4a, 100 pg/mL of rVvpM markedly increased the level of *Muc2* promoter methylation at the − 274 CpG site, but not at − 193 for 12 h. However, the level of *Muc2* promoter methylation at the − 274 CpG site was significantly inhibited by a treatment with NAC, siRNAs for *PKC*δ and *ERK*, and the DNA methylation inhibitor 5-azacytidine (5-Aza) (Fig. 4b). Interestingly, a pre-treatment with melatonin significantly inhibited the level of *Muc2* promoter methylation as induced by rVvpM, results which were abrogated by the aforementioned siRNAs for *MT*_*2*_*, Gαq* and *NCF-1* (Fig. 4c). In addition, a pre-treatment with 5-aza significantly blocked the repression of Muc2 evoked by rVvpM (Fig. 4d). These data indicate that melatonin acting on MT_2_ inhibits the ERK-mediated hypermethylation of the *Muc2* promoter to restore the level of Muc2 production in rVvpM-treated HT29-MTX cells.

### Melatonin restores Muc2 depletion induced by VvpM in a mouse ileum infected with *V. vulnificus*

**Fig. 5:**
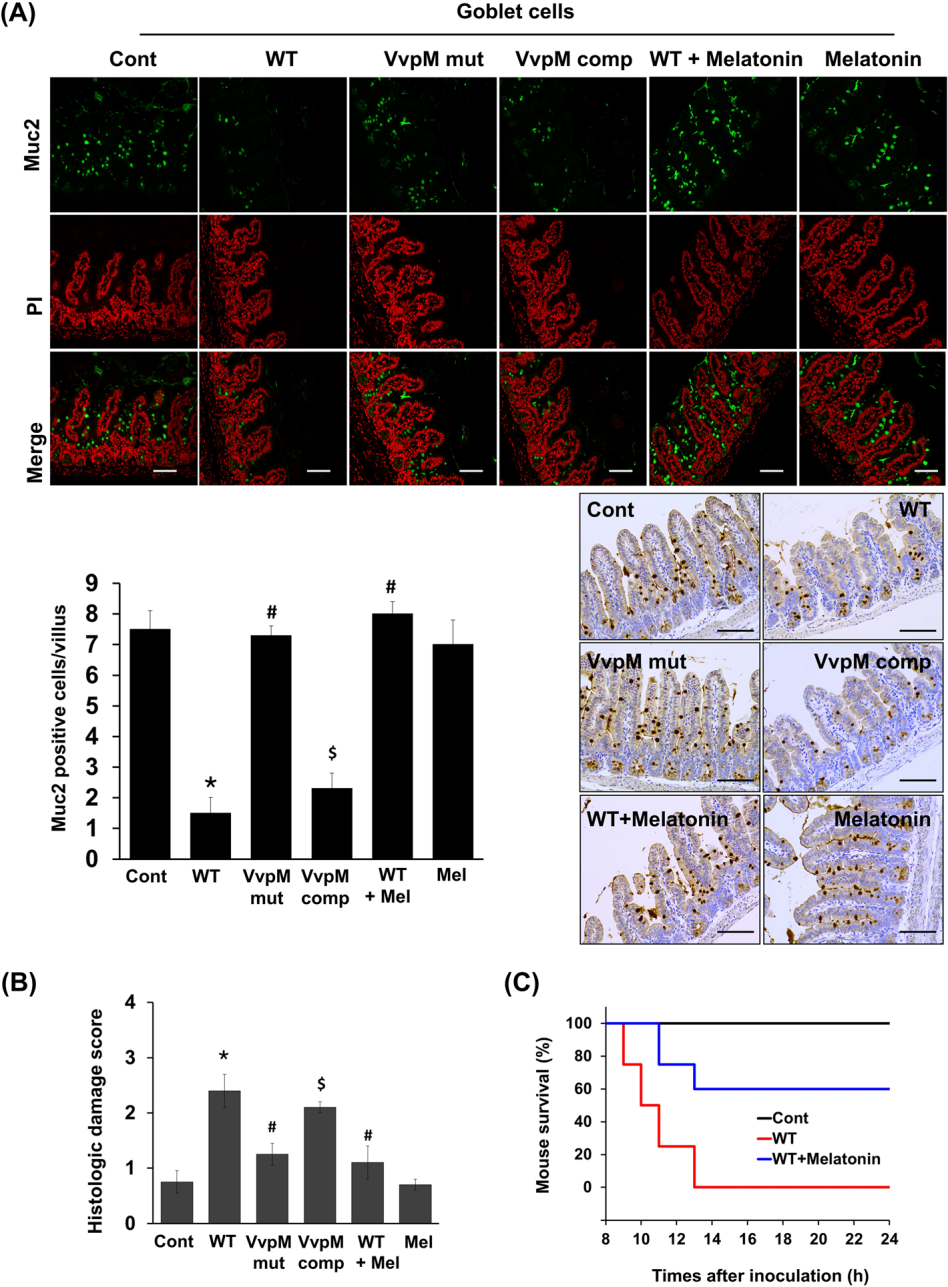
Melatonin restores Muc2 depletion induced by VvpM in mouse ileum infected with *V.vulnificus*. Mice inoculated with boiled *V. vulnificus* (Cont), *V. vulnificus* (WT), VvpM mutant (mut), and VvpM complement (comp) at 1.3 × 10^9^ CFU/mL, and sacrificed 16 h later. Mice were also given oral administration of melatonin (10 mg/kg) for 7 days prior to infection of *V. vulnificus* (WT). **a** Expression of Muc2 in mouse ileum was examined by immunofluorescence (Top panel, green) and immunohistochemical analysis (bottom panel, brown). Propidium iodide (PI, red) was used for nuclear counterstaining for immunofluorescence analysis. Scale bars, 100 μm. The mean numbers of Muc2-labeled cells per villi are shown in the bar graph. Data represent means ± S.E. *n* = 8. *, *p* < 0.01 vs. Cont. ^*#*^, *p* < 0.001 vs. WT. ^$^, *p* < 0.05 vs. VvpM mut. **b** Average scores of histopathologic damage index from mouse ileum are shown. Mel, melatonin. *n* = 8. *, *p* < 0.01 vs. Cont. ^*#*^, *p* < 0.01 vs. WT. ^$^, *p* < 0.051 vs. VvpM mut. **c** Iron-overloaded ICR mice received an oral administration of melatonin (10 mg/kg) daily for 7 days prior to i.p. inoculation with boiled *V. vulnificus* (Cont) and *V. vulnificus* (WT) at 1.2 × 10^2^ CFU/ml. Survival rate of mice treated with melatonin relative to WT is shown *n* = 10. *p* < 0.05

To evaluate the clinical relevance of *V. vulnificus* VvpM, we investigated the effect of VvpM on the expression of intestinal Muc2 in a mouse inoculated intragastrically with a control, WT, and with the VvpM mutant (mut) and VvpM complementation (comp) at 1.3 × 10^9^ CFU/mL for 16 h. Mice were also given an oral administration of melatonin (10 mg/kg) for seven days prior to infection of *V. vulnificus* (WT) for 16 h (Fig. 5a). The number of goblet cells immunostained by Muc2 in the ileum infected with WT was decreased by 6.0 ± 1.5/villus at 16 h compared to the control. However, when mice were inoculated with the mutant deficient in the *VvpM* gene (VvpM mut), the levels were increased by 5.8 ± 0.9/villus compared to those associated with the WT. In contrast, complementation of the VvpM mutant with a functional *VvpM* gene (VvpM comp) completely restored the effect of WT on the immunofluorescence staining of Muc2. These results indicate that VvpM plays an important role in the repression of Muc2 during a *V. vulnificus* infection. Importantly, the numbers of goblet cells decreased by WT infection were significantly recovered in the mice group pretreated with 10 mg/kg melatonin, suggesting that melatonin restores Muc2 depletion induced by VvpM in the mouse ileum infected with *V. vulnificus*. The functional role of melatonin on mucin repression induced by WT was further confirmed by the immunohistological staining of Muc2 (Fig. 5a, bottom panel). There were no significant differences between the numbers of goblet cells in mice treated with a control or with 10 mg/kg of melatonin alone, suggesting that exogenous melatonin at a concentration of 10 mg/kg does not have a relevant effect on the expression of intestinal Muc2 in mice. WT caused severe necrotizing enteritis of the intestine, where it induces shortened villi heights accompanied by an expanded width and increased inflammation at 16 h post oral infection, resulting in an increased histopathological damage score compared to that of the control mice (Fig. 5b). However, we found that an oral administration of the VvpM mutant failed to elevate the histopathological damage score caused by the WT infection (Fig. 5b). The functional effects of the VvpM mutant were significantly restored by treatment with VvpM complamentation (comp). Notably, an oral administration of 10 mg/kg of melatonin in WT mice decreased the histopathological damage score, showing nearly complete restoration of the damage by WT and suggesting that melatonin is a potential therapeutic for *V. vulnificus* infections because it maintains the physiological function during mucin production of intestinal goblet cells. We also investigated whether melatonin prevents lethal activity induced by *V. vulnificus.* We used an iron-overloaded mouse model quickly to bring the growth of *V. vulnificus* to the lethal level [35]. As shown in Fig. 5c, all of the mice injected with *V. vulnificus* were dead by 13 h post injection, whereas six of the ten mice pre-treated with melatonin remained alive for more than 24 h, showing some degree of the attenuation of mouse lethality due to the treatment with melatonin. This result indicates the severity of the infection of *V. vulnificus* and the clinical potential of melatonin.

## Discussion

Our data indicate that melatonin coupling with MT_2_ restores Muc2 depletion induced by *V. vulnificus* VvpM and that melatonin plays an important role in the inhibition of *Muc2* promoter hypermethylation occurring due to ROS production. These results are in contrast to the previously characterized effect of melatonin on mouse asthmatic conditions, where melatonin effectively attenuates mucus hypersecretion via a reduction in the MUC5AC expression level [19]. We believe that these broad and often contradictory effects of melatonin are in part due to the presence of the different mucin isoforms in different types of cells/tissue, and outcomes may vary depending on the cellular concept. In contrast to the lung tissue harboring abundant expressions of MUC5, the major component of the intestinal mucus layer is Muc2 in the human small intestine and colon, where it plays an important role as a physiological barrier via the formation of an enormous net-like mucus polymer barrier [2]. Indeed, *Muc2* deficiency in mice, which lack an inner mucus layer, causes the spontaneous development of inflammation, gross bleeding and increased paracellular permeability due to unusual commensal bacteria colonization [3, 4]. In addition, we have shown that Muc2 is the major intestinal *O*-glycosylated protein produced by goblet cells and HT29-MTX cells [33]. Concerning the concentration of melatonin, it was previously reported that the physiological levels of melatonin are in the picomolar and nanomolar ranges in the blood, whereas the melatonin concentrations of some tissues and cells can be exceptionally high. However, this was found to be physiological [36]. Given that the intestine is the main organ involved in the uptake of nutrients [37], the concentration of melatonin in the intestine could be high due to the melatonin present in several foods, including meat and vegetables. In addition, it was shown that cells in the gastrointestinal tract contain melatonin in high concentrations [38], indicating that melatonin in other body fluids and cells is not necessarily in equilibrium with those in the blood. Thus, we suggest that melatonin at 1 μM has a physiological function in the regulation of intestinal MT_2_ activation and Muc2 production during an infection with *V. vulnificus*. This means that the cellular mechanism of melatonin acting on the melatonin receptor to manipulate mucin production in the gastrointestinal tract differs from that of asthma and COPD in the lung.

In the present study, we presented several novel findings pertaining to the functional role of VvpM and melatonin based on at least two observations. First, our data demonstrate that *V. vulnificus* VvpM is the functional zinc-metalloprotease responsible for Muc2 repression in the mouse and that VvpM, acting through the ROS-dependent activation of the PKCδ/ERK pathway, inhibits *Muc2* expression by stimulating the methylation of the *Muc2* gene promoter in mucus-secreting HT29-MTX cells. In contrast to earlier work showing that many enteric pathogens directly degrade intestinal mucin by producing Pic [39], StcE [40], or Hap [41], we suggest that *V. vulnificus* regulates intestinal Muc2 expression levels by producing VvpM via a transcriptional regulatory mechanism with modes of action that differ from those of other enteric pathogens. Concerning the biological significance of the redundant Muc2 inhibition by VvpE and VvpM during a *V. vulnificus* infection, it was previously reported that VvpE makes a significantly faster contribution to Muc2 repression together with epithelial tissue damage [33]. However, the effect of rVvpM is not limited to Muc2 repression, as this toxin also shows a unique potential to disrupt the innate immune system by killing the recruited phagocytes that would promote rapid in vivo *V. vulnificus* growth and colonization [15]. Thus, it is possible that the combination of rapid growth and tissue damage together with the Muc2 repression evoked by VvpE and VvpM is one mechanism that promotes dissemination to the bloodstream during the *V. vulnificus* infection cycle. In addition, it clear that the impairment of the mucus layer enhances the colonization of pathogens with the establishment of an appropriate portal of entry, where it gains access more easily to epithelial cells and thereby promotes pathogen-host adherence mechanisms. Thus, these results suggest VvpM plays an important role in the pathogenesis of *V. vulnificus* in the gastrointestinal tract. Second, we are the first to show that HT29-MTX cells pretreated with melatonin acting through MT_2_ have the ability to block Muc2 depletion induced by rVvpM. Thus, goblet cells may be more protective against an infection of *V. vulnificus* if they are pre-activated by melatonin. The pre-activation of HT29-MTX cells may offer a means by which to improve the protective potency of these cells without the need for an additional treatment of antibiotics to eliminate *V. vulnificus*. Given that MT_2_ expression is most abundant in HT29-MTX cells and considering that the loss of the MT_2_ function significantly blocks rVvpM-induced mucin repression, our results indicate that MT_2_ is the major melatonin receptor that regulates the signaling pathway responsible for mucin production, thus providing the first evidence that melatonin may have a unique MT_2_ receptor signaling pathway during a *V. vulnificus* infection. Therefore, our findings suggest that melatonin is a good candidate for host cell protection against *V. vulnificus* infections when used as a pre-activation agent that induces the MT_2_ signaling pathway.

The MT_2_ receptor initially couples with heterotrimeric Gα proteins. It was shown previously that MT_2_ couples with mainly Gαi and Gq, whereas MT_1_ interacts with Gαi, Gαq, and Gαs [29, 42, 43]. In HT29-MTX cells treated with rVvpM, however, we found that mucin production mediated by MT_2_ was selectively regulated by Gαq, not by Gαi or Gα12. These results are supported by those in a previous study in which Gαq subunit-linked signal transduction was found to be a critical step in the regulation of melatonin signaling to block the apoptotic and autophagic cell death induced by *V. vulnificus* in intestinal epithelial cells [44]. Moreover, we noted that melatonin blocks ROS production via MT_2_ acting with NCF-1 in HT29-MTX cells treated with rVvpM. The ROS generated by a bacterial infection have been shown to induce the oxidative inactivation of several proteins harboring oxidant-sensitive thiol groups and of the ubiquitin–proteasome pathway [45], thereby activating many redox-sensitive proteins, including regulators of MAP kinase pathways [46]. NADPH oxidase including p47 ^phox^ (NCF-1) is a critical source of cellular ROS and has been studied extensively in phagocytes as an innate host defense component [47, 48]. Increasing evidence has suggested that lipid rafts are clustered to form a redox-signaling platform through the activation of NOX2 by interacting with NCF1 and Rac1 for membrane ROS production [47, 49]. However, it has been shown that melatonin signaling via MT_2_ triggers the recruitment of NCF-1 into non-lipid rafts to block ROS production induced by *V. vulnificus* in intestinal epithelial cells [44]. Thus, our results here suggest that melatonin is a functional agent preventing the virulence effect of rVvpM by regulating the spatial distribution of MT_2_ and NCF-1 to block ROS production during the regulation of bacterial infections.

We subsequently showed that melatonin has an inhibitory effect on the phosphorylation of PKCδ responsible for ERK activation to block mucin depletion as induced by rVvpM. Many bacterial stimuli regulate the PKC and MAPK pathways, both of which are interesting candidates as downstream mediators of ROS. For instance, it was previously revealed that the presence of *H. pylori* induces the phosphorylation of a novel type of PKCδ to activate ERK kinase pathways [50], whereas an *H. pylori* infection also regulates p38 MAPK activation to promote the ROS signaling pathway [51]. However, other authors showed that conventional PKCα activation was found to be necessary for the impairing of the intestinal barrier function induced by an *Enteropathogenic E. coli* infection [52]. These results indicate that the cellular pathways activating PKC and MAPK differ in terms of the type of bacterial pathogen. Indeed, we previously demonstrated that *V. vulnificus* activates PKCδ and ERK via ROS production during the prompting of mucin repression [19], suggesting that rVvpM selectively regulates specific PKC isozymes and MAPK phosphorylation. On the other hand, PKC and MAPK pathways mediated by melatonin receptor coupling with Gαq play critical roles in the regulation of the circadian effects of melatonin [16]. Hence, it is conceivable that melatonin has a potential role in Muc2 depletion induced by rVvpM via the regulation of PKCδ /ERK cascades. Therefore, our results strongly suggest that melatonin is a functional agent that blocks ROS-dependent PKCδ/ERK activation during the promotion of mucin recovery in goblet cells infected with *V. vulnificus*. In addition, having shown that rVvpM triggers the signaling cascade for ROS production as well as PKCδ and ERK phosphorylation to ameliorate Muc2 production in a short period of time (within 15 min), we suggest that a pre-treatment has an advantage in that the melatonin reaches a steady state to concert with the MT_2_ signaling pathway, which induces the recruitment of NCF-1 into non-lipid rafts to prevent the production of ROS in the membranes of intestinal epithelial cells [44]. On the other hand, there are potential problems with therapeutic/suppressive agents when eliminating bacteria itself owing to the growing problem of antibiotic resistance. However, we suggest that melatonin is a unique antibiotic-free agent that manipulates the foodborne pathogen signaling pathway by preventing the clustering of the redox-signaling platform in the membranes of intestinal epithelial cells.

Next, we attempted to uncover the mechanism by which the ROS/PKCδ/ERK pathway is linked to other molecular mechanisms that are closely related to mucin repression in cells treated with rVvpM. The promoter methylation of cytosine residues at CpG dinucleotides is an important epigenetic mechanism during the promotion of the transcriptional repression of *Muc2* [53]. Indeed, it was clearly shown that ROS influences global histone modification and DNA methylation, which are key events responsible for the expression of genes [54]. Compelling evidence further supports the role of PKCδ and ERK during the hypermethylation of tumor-suppressor genes and the pathogenesis of colon cancer [34]. Our current results indicate that rVvpM induces the region-specific methylation of the *Muc2* promoter at the − 274 site through ERK, suggesting that this bacterial pathogen promotes the hypermethylation of the *Muc2* promoter to repress *Muc2* expression. In contrast, we found that the inhibition of DNA methylation by melatonin blocks rVvpM-induced Muc2 depletion. Given that melatonin inhibits the ROS/PKCδ /ERK pathway responsible for mucin repression, we suggest that the inhibitory effect of melatonin on the *Muc2* promoter hypermethylation is likely to be compounded by the selective expression of MT_2_ coupling with Gαq in HT29-MTX cells. Thus, the results here indicate that melatonin regulates the distinctive infectious stratagems of *V. vulnificus* to control mucin gene expression by manipulating the host methylation status.

Finally, in the mouse models of *V. vulnificus* infection, our results obtained from gain- and loss-of-function approaches for VvpM revealed that VvpM is an important virulence factor of *V. vulnificus* responsible for mucin depletion. These results are further supported by a previous study in which VvpM was found to contribute to *V. vulnificus* colonization, facilitating pro-inflammatory responses in the mouse intestine [44]. Thus, our result indicates that *V. vulnificus* uniquely subverts the host signal pathways by secreting VvpM with different modes of infection mechanisms to circumvent the host defensive responses in the gut. Importantly, we also prove here that melatonin blocks the mucin depletion of goblet cells infected with *V. vulnificus*, suggesting that the functional role of melatonin of neutralizing the bacterial toxin activity involved in Muc2 repression may provide potential therapeutic strategies for bacterial pathogen infections in the intestinal epithelium. This means that melatonin therapy could aid in the development of new therapeutic strategies to control bacterial infections, ultimately providing deeper insight into various intestinal disorders.

## Conclusion

Overall, these findings highlight the relevance of the melatonin coupling with the MT_2_ signaling pathway during Muc2 depletion induced by *V. vulnificus* VvpM and provide important insight into the potential for the development of therapeutic strategies and agents to treat *V. vulnificus* infections. Although our study demonstrated that a pretreatment of melatonin onto goblet cells leads to significantly improved mucin production during a *V. vulnificus* infection while also defining the molecular mechanism by which melatonin acts during this process, further research is required to establish in greater detail in the effect of melatonin on the mucin production process.

## Supplementary information

**Additional file 1: Table S1.** Oligonucleotides used in this study. **Table S2.** Primers used in Muc2 methylation analysis. **Figure S1.** Effect of rVvpM on cell viability and Muc2 production. (A) HT29-MTX cells were incubated with 100 pg/ml of rVvpM for 24 h, and the viability of cells was measured. Data represent means ± S.E. *n* = 3. (B) Time responses of rVvpM in expression of Muc2 are shown. Data represent means ± S.E. *n* = 4. *, *p* < 0.01 vs. 0 h. ROD, relative optical density. **Figure S2.** Melatonin regulates the level of Muc2 in intestinal epithelial cells treated with rVvpE. (A) HT29-MTX cells were treated with melatonin (1 μM) for 30 min prior to rVvpE (50 pg/mL) exposure for 4 h. The level of Muc2 protein was quantified by ELISA. Data represent means ± S.E. *n* = 3. *, *p* < 0.01 vs. vehicle. ^*#*^, *p* < 0.05 vs. rVvpE alone. **Figure S3.** Effect of rVvpM on the levels of production of ROS and phosphorylation of PKCδ and ERK. (A) HT29-MTX cells were incubated with 100 pg/ml of rVvpM for 12 h, and the production of ROS was measured. RFU, relative fluorescence units. Time responses of rVvpM in phosphorylation of PKCδ (B) and ERK (C) are shown. Data represent means ± S.E. *n* = 4. *, *p* < 0.05 vs. 0 h. ROD, relative optical density.

## Data Availability

The datasets used and/or analysed during the current study are available from the corresponding author on reasonable request.

## Abbreviations

CM-H_2_DCFDA: 2′, 7′ -dichlorofluorescein diacetate
ERK: Extracellular signal-regulated kinase
IL-1β: Interleukin-1β
MAPKs: Mitogen-activated protein kinases
MT_2_: Melatonin receptor 2
Muc: Mucin
NCF1: Neutrophil Cytosolic Factor 1
PKCδ: Phosphorylation of protein kinase C δ
ROS: Reactive oxygen species
*Vibrio vulnificus*: *V. vulnificus*
WT: Wild Type
