# Supplementary material for: Melatonin restores Muc2 depletion induced by V. vulnificus VvpM via melatonin receptor 2 coupling with Gαq
Source: J Biomed Sci. 2020 Jan 6;27:21. doi: 10.1186/s12929-019-0606-x (PMC6943958; doi:10.1186/s12929-019-0606-x)
Supplement: Supplementary file 1 — Additional file 1: Table S1. Oligonucleotides used in this study. Table S2. Primers used in Muc2 methylation analysis. Figure S1. Effect of rVvpM on cell viability and Muc2 production. (A) HT29-MTX cells were incubated with 100 pg/ml of rVvpM for 24 h, and the viability of cells was measured. Data represent means ± S.E. n = 3. (B) Time responses of rVvpM in expression of Muc2 are shown. Data represent means ± S.E. n = 4. *, p < 0.01 vs. 0 h. ROD, relative optical density. Figure S2. Melatonin regulates the level of Muc2 in intestinal epithelial cells treated with rVvpE. (A) HT29-MTX cells were treated with melatonin (1 μM) for 30 min prior to rVvpE (50 pg/mL) exposure for 4 h. The level of Muc2 protein was quantified by ELISA. Data represent means ± S.E. n = 3. *, p < 0.01 vs. vehicle. #, p < 0.05 vs. rVvpE alone. Figure S3. Effect of rVvpM on the levels of production of ROS and phosphorylation of PKCδ and ERK. (A) HT29-MTX cells were incubated with 100 pg/ml of rVvpM for 12 h, and the production of ROS was measured. RFU, relative fluorescence units. Time responses of rVvpM in phosphorylation of PKCδ (B) and ERK (C) are shown. Data represent means ± S.E. n = 4. *, p < 0.05 vs. 0 h. ROD, relative optical density. [file 12929_2019_606_MOESM1_ESM.docx]

**Additional file 1**

**Melatonin restores Muc2 depletion induced by *V. vulnificus* VvpM via melatonin receptor 2 coupling with Gαq**

Young-Min Lee^1^, Jong Pil Park^1^, Young Hyun Jung^2^, Hyun Jik Lee^2^, Jun Sung Kim^2^, Gee Euhn Choi^2^, Ho Jae Han^2^*, Sei-Jung Lee ^1^*

**Table S1. Oligonucleotides used in this study**

| **Name** | **Oligonucleotide Sequence (5' → 3')a, b** | | **Use** | |
| --- | --- | --- | --- | --- |
| **For mutant construction** | | | | |
| VvpM-upF | | CGTGACATTTTGGGCCCTCTAGT | | Deletion of *VvpM* ORF |
| VvpM-upR | | CGGGATCCCCGACATCATGGGCACCAACAT | |  |
| VvpM-downF | | CGGGATCCCATTTTGTTCCCACCATAGG | | Deletion of *VvpM* ORF |
| VvpM-downR | | CGAGCTCGGTGAACCTTAAGATGGCGC | |  |
| **For mutant complementation** | | | | |
| VvpM001F | | GGATCCAGTTTTGCAATCCTATGGTG | | Amplification of the *VvpM* ORF |
| VvpM001R | | GAGCTCCTAGTTTTGTGAACAAAAGG | |  |

^a^ The oligonucleotides were designed using the *V. vulnificus* MO6-24/O genomic sequence (GenBank^TM^ accession number CP002469 and CP002470, www.ncbi.nlm.nih.gov).

^b^ Regions of oligonucleotides not complementary to the corresponding genes are underlined.

**Table S2. Primers used in Muc2 methylation analysis.**

| **CpG site in Muc2 promoter region** | |  | | **Identification** | | | **Primer sequence, 5’–3’** | **Size**  **(bp)** | |
| --- | --- | --- | --- | --- | --- | --- | --- | --- | --- |
|  |  | |  | |  | | | |  |
| *-274* | Methylated | | Sense  Antisense | | GTTTATGGYGGGTTAAGGAGTTTGAT  ACCCGAAAAACACATACAACTACTAAAAAAACG | | | | 105 |
|  | Unmethylated | | Sense  Antisense | | GTTTATGGYGGGTTAAGGAGTTTGAT  ACCCAAAAAACACATACAACTACTAAAAAAACA | | | | 105 |
|  |  | |  | |  | | | |  |
| *-193* | Methylated | | Sense  Antisense | | ATAGGGTTGTTTTATTTTGAAGAAGGTTGC  AACATCTACCAAATAATCAAAAAAACAACTA | | | | 137 |
|  |  | |  | |  | | | |  |
|  | Unmethylated | | Sense  Antisense | | ATAGGGTTGTTTTATTTTGAAGAAGGTTGT  AACATCTACCAAATAATCAAAAAAACAACTA | | | | 137 |
|  |  | | |  | |  | | |  |

**
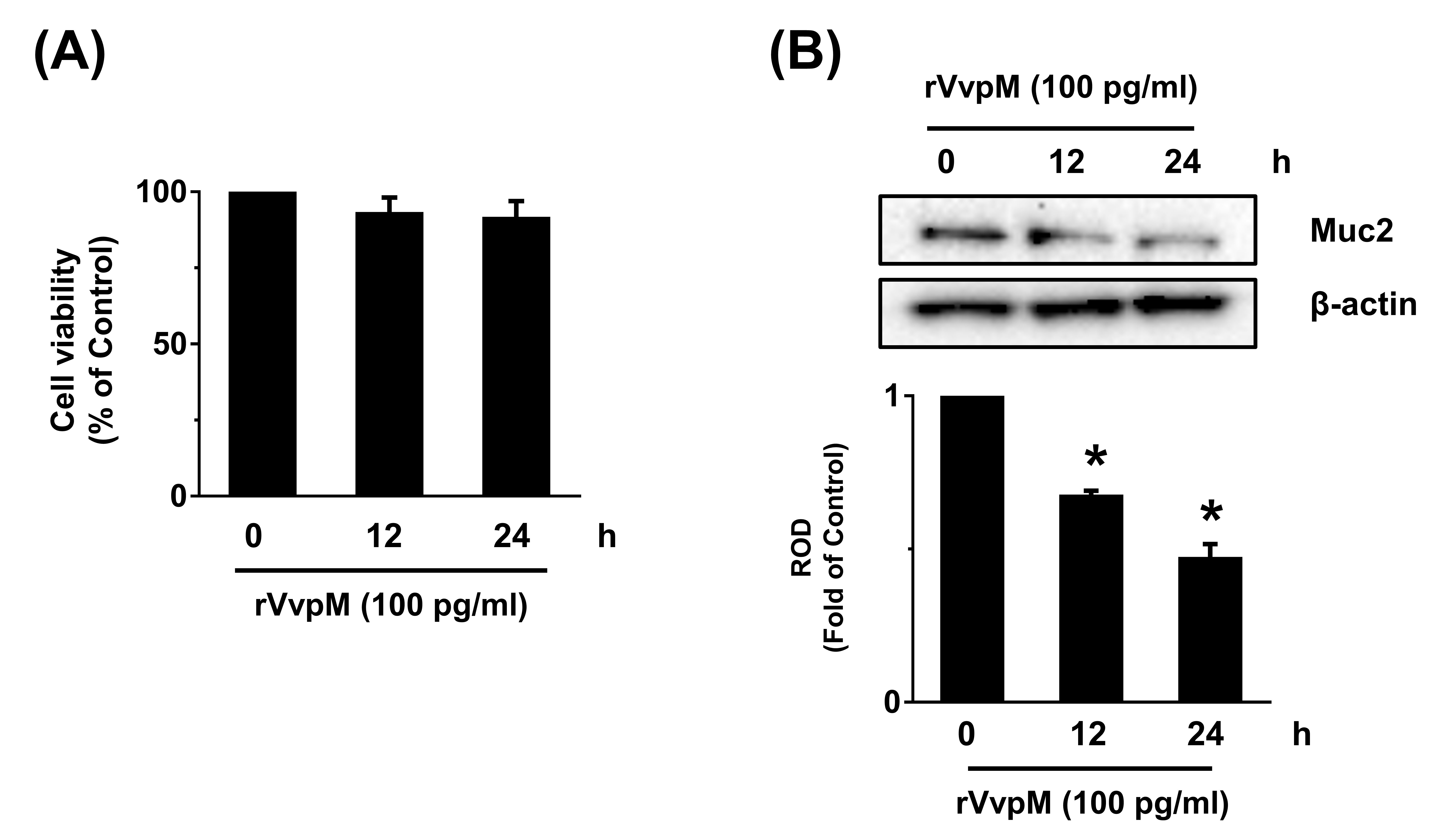
**

**Figure S1. Effect of rVvpM on cell viability and Muc2 production.** (A) HT29-MTX cells were incubated with 100 pg/ml of rVvpM for 24 h, and the viability of cells was measured. Data represent means ± S.E. *n* = 3. (B) Time responses of rVvpM in expression of Muc2 are shown. Data represent means ± S.E. *n* = 4. *, *p* < 0.01 vs. 0 h. ROD, relative optical density.


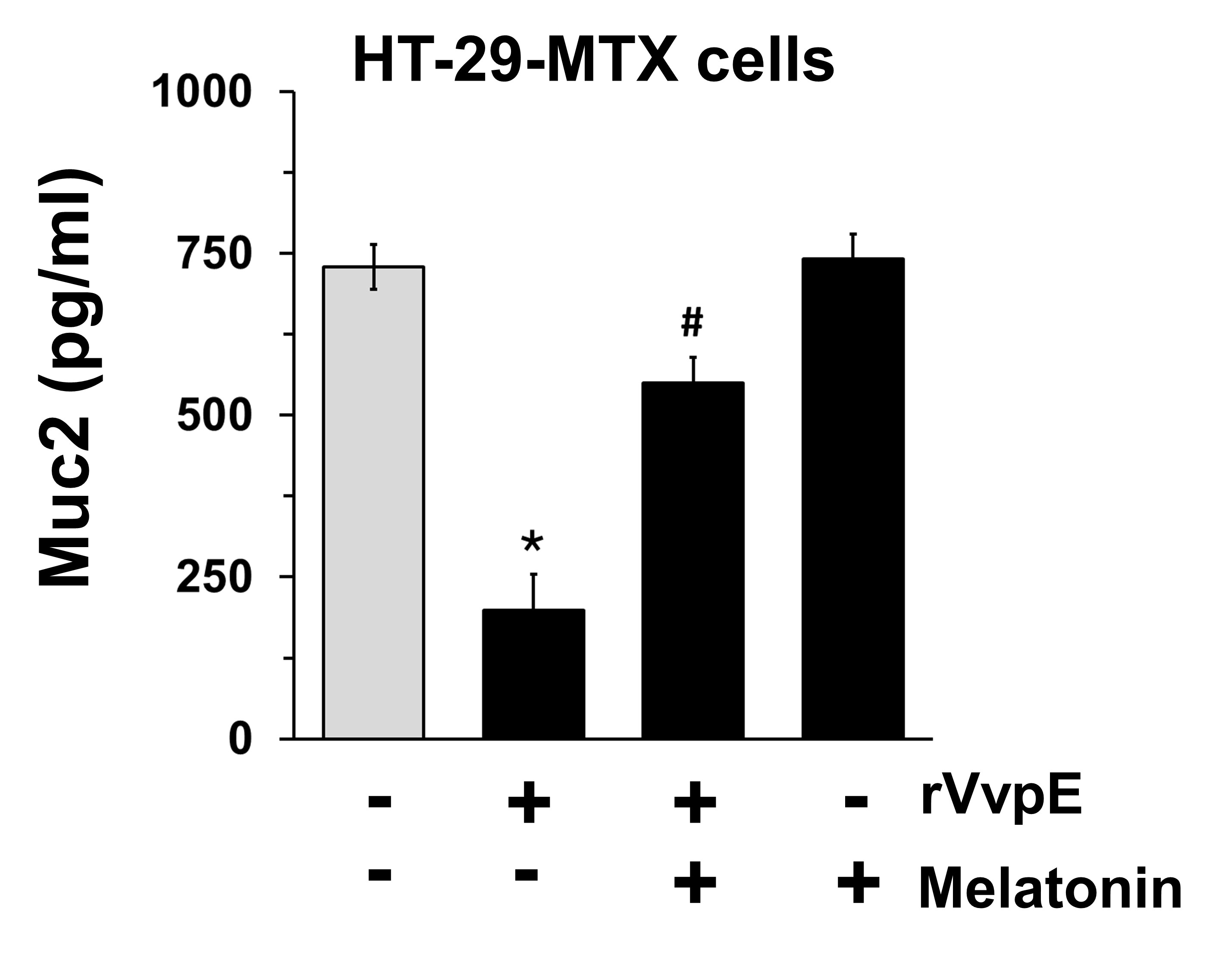


**Figure S2. Melatonin regulates the level of Muc2 in intestinal epithelial cells treated with rVvpE.** (A) HT29-MTX cells were treated with melatonin (1 µM) for 30 min prior to rVvpE (50 pg/mL) exposure for 4 h. The level of Muc2 protein was quantified by ELISA. Data represent means ± S.E. *n* = 3. *, *p* < 0.01 vs. vehicle. *^#^*, *p* < 0.05 vs. rVvpE alone.


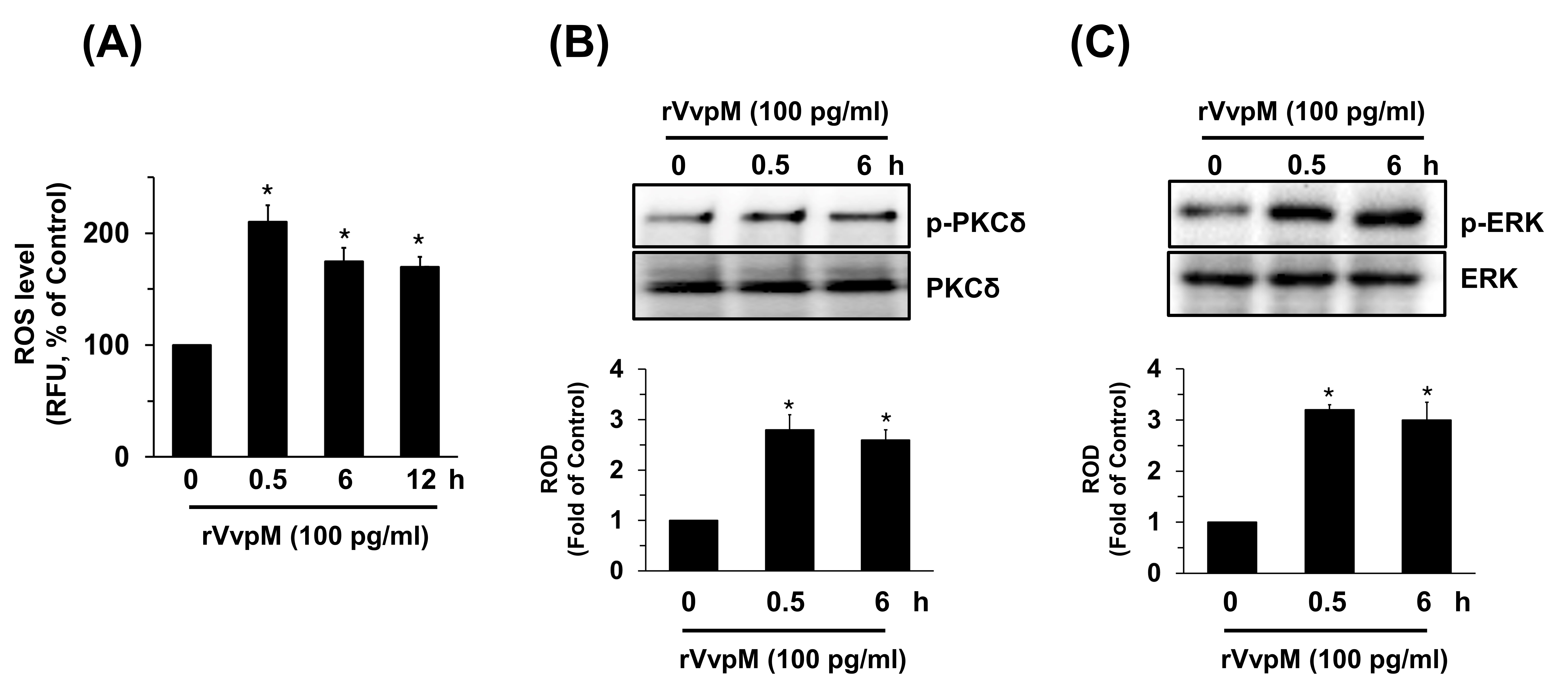


**Figure S3. Effect of rVvpM on the levels of production of ROS and phosphorylation of PKCδ and ERK.** (A) HT29-MTX cells were incubated with 100 pg/ml of rVvpM for 12 h, and the production of ROS was measured. RFU, relative fluorescence units. Time responses of rVvpM in phosphorylation of PKCδ (B) and ERK (C) are shown. Data represent means ± S.E. *n* = 4. *, *p* < 0.05 vs. 0 h. ROD, relative optical density.
